# Livestock predation, crop raiding, and community attitudes towards sustainable wildlife conservation in and around Mankira Forest, Southwest Ethiopia

**DOI:** 10.1186/s12862-024-02279-2

**Published:** 2024-06-27

**Authors:** Birhanu Asaye, Wondimagegnehu Tekalign, Taye Dobamo

**Affiliations:** Natural and Computational Sciences College, Biology Department, Wolaita Sodo University, PO Box 138, Wolaita Sodo, Ethiopia

**Keywords:** Crop raiders, Domestic animal predation, Negative attitude, Wildlife conservation

## Abstract

Crop raiding and livestock predation negatively impact the views of the local community towards wildlife conservation. Farmers across the African continent, especially those in rural regions, incur financial losses as a result of crop raiding and livestock depredation. The sustainability of the forest relies heavily on comprehending the essential connection between a harmonious park-people relationship and the coexistence of humans and wildlife. The primary aim of this study is to evaluate the predation of livestock, the raiding of crops, and the attitudes of the community towards wildlife in the Mankira Forest located in southwest Ethiopia. This particular area has been lacking in scientific research, making it crucial to conduct this assessment. The data were collected between November 2021 and September 2022 via a structured questionnaire. This study used a sample of 241 randomly selected respondents from the four villages, and responses were compared using chi-square tests. Pearson correlation was also used to test the relationship between the distance of farmland and the extent of crop raiding. The majority of the respondents (95%) reported the presence of crop raiding and livestock predation in the area. These losses were caused by the *Papio anubis* (39%), the *Chlorocebus aethiops* (24.1%), the *Hystrix cristata* (15.3%), the *Canis aures* (58.3%), and the *Crocutacrocuta* (29.5%). Maize stood out as the crop type most susceptible to crop raiders. Most of the respondents (56.7%) had a negative attitude towards wildlife conservation. There was a significant difference among age groups of respondents related to their attitude towards wildlife conservation (*p* < 0.05). The study highlights the need to address several gaps in understanding and managing human-wildlife conflict through research on predation, raiding, and community attitudes. Therefore, to fulfill the dual goals of community support and conservation of wildlife, rigorous management and planning are needed.

## Introduction

Livestock predation and crop raiding by wildlife have a major economic impact on the African continent, especially on rural communities [1, 4, 9, 10, 24, 25, 32, 44, 46]. Developing countries are more susceptible to crop raiding and livestock depredation because their economies depend heavily on the subsistence use of natural resources [10, 26], and their livelihoods depend on agriculture and raising livestock [17, 20, 22, 25, 35, 43, 47, 50].

Community livelihoods, attitudes, and tolerance towards wildlife and its habitats are impacted by the economic losses [22, 35, 38, 46]. It also fosters negative attitudes towards the importance of wildlife species conservation [38, 52]. It is currently a common occurrence and a problem for conservationists globally [26, 35, 46]. Particularly, it has been demonstrated that crop raiding by wild animals and livestock predation hinder conservation efforts [4, 15, 16, 36, 48]. If the damage significantly affects the norms of living for the affected people, it will be difficult to obtain their active support for conservation efforts [4].

Understanding the complex dynamics of interactions between humans and animals is crucial to ensuring enhanced coexistence and the conservation of wildlife species. Since human populations and wildlife habitats frequently overlap in Ethiopia, there are serious problems with crop raiding and livestock depredation by wildlife. In rural areas, where communities primarily depend on crops and livestock for their livelihoods, the situation becomes particularly serious. Even so, there are still a number of areas in which our knowledge of the problem is lacking, including long-term monitoring, socioeconomic factors, community involvement and livelihood diversification, and ecological effects. In order to ensure that humans and animals cohabit in shared environments and to reduce human-wildlife conflict in Ethiopia, it will be imperative to address these knowledge gaps.

In order to develop forest management plant for the study area, it is crucial to gather information about the extent of damage and its impacts. Restoring the balance between wildlife and farmers is imperative. It’s also important to assess the local community’s perspective on conserving animals. On the other hand, there has been no scientific research conducted in or around Mankira Forest regarding the aforementioned challenges of human-wildlife coexistence. It’s critical to identify the wild animals causing damage to livestock and crops, report them to the relevant authorities, and investigate any possible mitigation measures. Therefore, the purpose of this study was to collect fundamental scientific data regarding livestock predation, crop raiding, and locals’ attitudes towards wildlife.

## Materials and methods

### Study area description

**Fig. 1:**
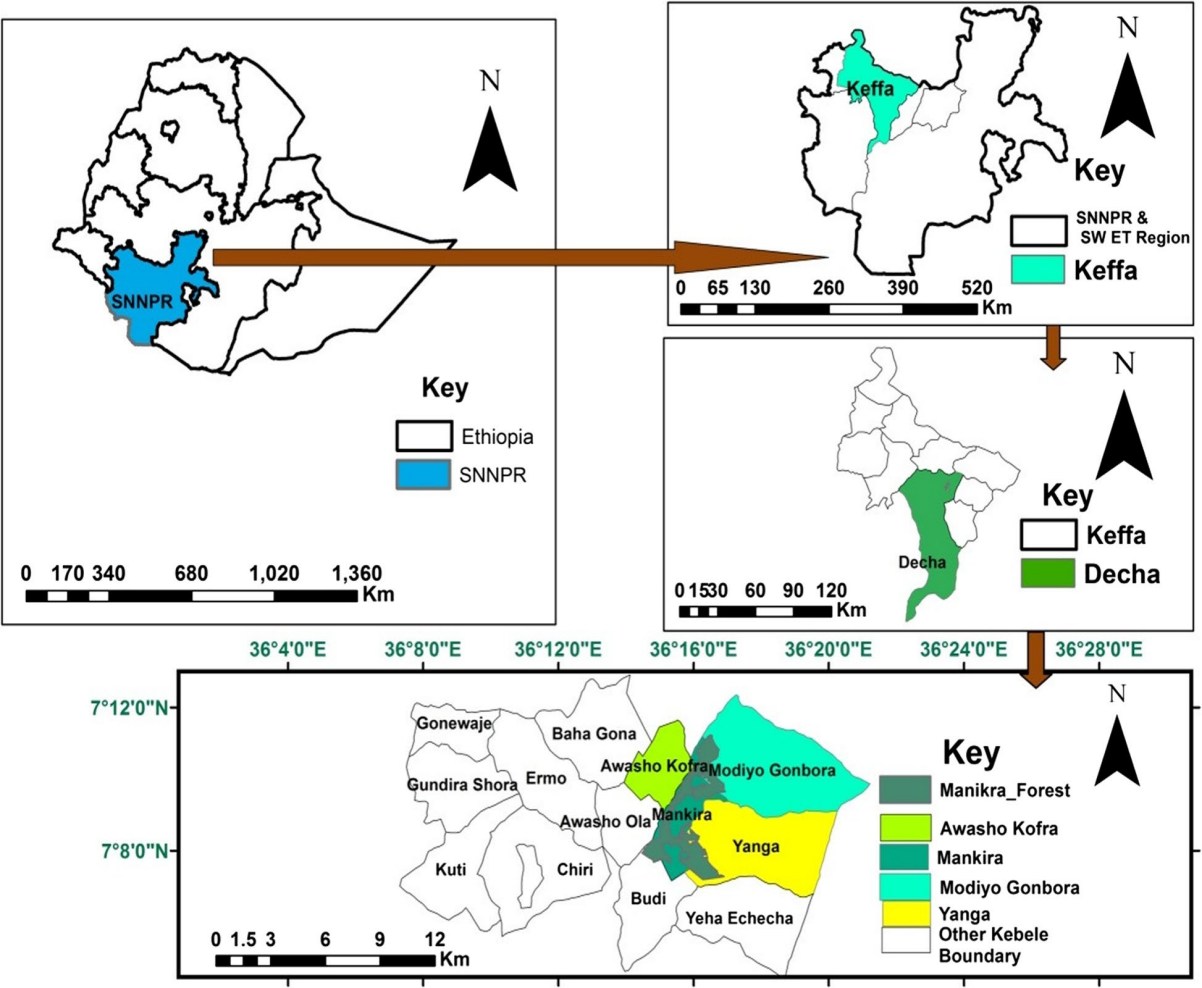
Location map of the study area. (source: Arc GIS)

Mankira Forest was purposively selected for this study because the people who live close to the forests are most affected by crop raiding and livestock losses. Mankira Forest is found in Kaffa Zone, Decha District, South West People’s Regional State, Southern Ethiopia. It is situated between 07^o^6’ and 07^o^10’N latitude and 36^o^6’ and 36^o^20’E longitude (Fig. 1). The forest is 461.5 km away from Addis Ababa and 12.5 km from Bonga Town, the capital city of Kaffa Zone.

The forest covers about 840.88 hectares and is surrounded by six *kebeles* (the smallest administrative unit in Ethiopia), namely Budi bordering in the south, Awasho Kofira in the west, Awasho Olla in the southwest, Modio Gonbera in the north, Gedam to the northwest, and Yanga to the east. The forest is bordered by two rivers: the Gumi River in the west and the Atesho River in the northeast. The study area is characterized by rough topography with much plain, hills, slopes, gorges, and plateaus. It has an elevation of 2,446 m above sea level [42].

According to 14 years’ data (from 2006 to 2019) from ENMA [12], the mean annual rainfall in the study area was about 1328.7 mm, ranging from 1043.4 mm to 1519.1 mm. The area is characterized by a wet season from June to September and a short dry spell of showers from March to May. The annual rainfall pattern of the area is bimodal from June to September. There is a relatively dry season from November to the end of February. The mean annual maximum and minimum temperature records of the study area were 21 ^0^C and 18 ^0^C, respectively. The mean annual maximum temperature was 19.7 ^0^C in March, and the mean annual minimum temperature was 15.6 ^0^C in August.

The most dominant plant species of the forest are African juniper (*Juniperus procera*), Elgon teak (*Olea welwitschii*), coffee (*Coffea arabica*), *wanza* (*Cordia africana*), *bisana* (*Croton macrostachvus*), ‘endod’ or African soapberry (*Phytolacca dodecandra*), loganberry (*Rubuslogano baccous*), water pear (*Syzygium guineense*), *girawa* or bitter leaf (*Vernonia amygdalina*), some edible fruit trees (*Psidium guajava* L.), Figs (*Ficus sur*), Governor’s plum (*Flacourtia indica*), African caper (*Capparis tomentosa*), and different species of shrubs. Some of the faunal species of the forest area are: warthog (*Phacochoerus africanus*), leopard (*Panthera pardus)*, bush pig *(Potamochoerus larvatus)*, African civet (*Civettictis civetta)*, spotted hayena (*Crocuta crocuta)*, golden jackal (*Canis aures)*, Anubis baboon (*Papio anubis)*, porcupine (*Hystrix cristata)*, grivet monkey *(Chlorocebus aethiops)*, rapptor birds, and other diverse species of birds [11].

The livelihood of the majority of the local people is mixed farming, i.e., livestock rearing and crop production. The crops growing in the area are mainly: coffee (*Coffea Arabica)*, teff (*Eragrostis tef)*, wheat *(Triticum aestivum)*, maize (*Zea mays)*, barley *(Hordeum vulgare)*, common bean *(Phaseolus vulgaris)*, sorghum *(Sorghum bicolor)*, and pea (*Pisum sativum)*. Additionally, potatoes (*Solanum tuberosum)*, tomatoes *(Solanum lycopersicum)*, onions *(Allium cepa)*, beets *(Beta vulgaris)*, and cabbages *(Brassica oleracea)* are grown by irrigation. The livestock of the area includes cattle *(Bos taurus)*, sheep *(Ovis aries)*, goats *(Capra hircus)*, donkeys *(Equus africanus asinus)*, and horses *(Equus ferus caballus)*.

### Study design, sample size determination, sampling technique, and data collection

A preliminary survey was conducted by field observation in October 2021 for three days before the actual data collection started to gather basic information about the study area. The descriptive type of research method was applied in this study as it gave the answer to a wide range of ‘what’, ‘when’, and ‘how’ questions pertaining to a particular population or group. This type of method is applied to describe social events, structures, and situations [11]. The purposive sampling method was used to choose the villages of respondents that were involved in the data collection. This allowed us to describe the major impact the findings have on the population within the selected communities. A systematic random sampling technique was applied to select every third HH on the list of households that participated in the interviews, questionnaires, and collection of tangible information. The systematic sampling helps minimize biassed samples and poor survey results in the study.

To assess livestock depredation and crop raiding by wild animals, the study employed a questionnaire survey method among the villages. Among the seven communities surrounding the research area (Gola, Yetiti, Bahita, Becha, Gechana, Chega, and Arida), four villages (Becha, Yetiti, Bahita, and Gola) were specifically chosen due to their closeness to the forest and the greatest rates of conflict between humans and wild animals in the area. The villages were then divided into three categories according to how close they were to the forest edge: near (0.5–1 km), medium (1–3 km), and far (4–5 km). Twenty randomly chosen local people, not part of the main sample group, who were of different ages, sexes, and backgrounds from all four villages pretested the questionnaire. After getting the total number of households living in each of the selected villages (i.e., Gola 185 HH, Yetiti 124 HH, Bahita 183 HH, and Becha 152 HH, for a total of 644), the sample size was determined by using the formula of the probability sampling technique [8].

$${\text{n}}_{0}= \frac{{\text{z}}^{2 }\text{p}\text{q}}{{\text{d}}^{2}}{ \text{n}}_{0}=\frac{{\left(1.96\right)}^{2}\left(0.5\text{*}0.5\right)}{({0.05)}^{2}}=384$$

As the sample size exceeded 5% of the population size (5% of 644 = 33, which is less than 384), a finite population correction formula was applied (Table 1).

$${\text{n}}_{1}=\frac{{\text{n}}_{0}}{1+({\text{n}}_{0}/\text{N})}{ \text{n}}_{1}= \frac{384}{1+(384/644)} = 241$$

Where: ${\text{n}}_{0}$= desired sample size

n_1_ = finite population correction factors less than 10,000.

Z = standard normal deviation (1.96 for the 95% confidence interval).

*P* = 0.5 (estimated proportion of the population to be included in the sample, i.e., 50%).

D = degree of desired accuracy (0.05) and q = 1-p (i.e., 0.5).

*N* = total population of households (644).

The percent of sample size in each village was computed by using C =$\frac{\mathbf{n}}{\mathbf{N}}\times 100$%, where C = percent of sampled households, *n* = total number of selected sampled households, and *N* = total population in the four sample villages.

$$\mathbf{C}=\frac{241}{644}\times 100\mathbf{\%}=37.42\mathbf{\%}$$.

A comparable sample size among the four villages was determined in proportion to their household numbers by using a simple proportion formula adopted from Cochran [8]: n_i_ = n×$\frac{\varvec{N}\varvec{i}}{\varvec{N}}$, I = 1, 2, 3…, n_i_ = sample size of each village, N_i_ = total population size in each village, *n* = total sample size, *N* = total population size of the four villages.

Based on the above calculation, 241 respondents were sampled. After the sample sizes were determined, systematic random sampling techniques were used to select the respondents from the total population of 644. 69 HH from Gola, 46 HH from Yetiti, 69 HH from Bahita, and 57 HH from Becha were chosen for this study. The number of sample household heads assigned to each village is proportionate to the number of household heads residing in each selected village (Table  1).

**Table 1:** Sample size of the households from each village near the Mankira Forest

Village	Total number of households			The sample size			Percentage
	Male	Female	Total	Male	Female	Total	
Gola	172	13	185	64	5	69	28.63
Yetiti	110	14	124	41	5	46	19.09
Bahita	163	20	183	61	8	69	28.63
Becha	137	15	152	51	6	57	23.65
Total	582	62	644	217	24	241	100.00

Both closed-ended and open-ended pretested questions were included in the questionnaires. In order to facilitate accessible communication during data collection and minimize misunderstandings, the questions were prepared in English and translated into ‘Kafinoonoo’. The responses from the respondents were then translated back into English. The period of data collection was 2021–2022. Random selection was used to choose each respondent from the study village, with a pattern of missing two households before interviewing the third [14]. Twelve community members, three from each of the four research villages, were chosen and given training. The average duration of each interview was forty-three minutes, with a range of thirty to fifty minutes. Participants’ ages ranged from 18 to 65 and older. It promotes inclusivity, records a range of viewpoints, and offers insightful information on a number of aspects of human existence for people of all ages. The households were given the questionnaires at random, using a first-come, first-served approach. Four primary areas of interest were covered by the questionnaires: (i) demographic and socioeconomic data; (ii) the identification of problematic wild animals that cause crop raiding and domestic animal depredation; (iii) the extent of raided crops and depredated livestock; and (iv) farmers’ attitudes towards wild animal conservation by applying the approaches of Naughton-Treves and Treves [39]; Gebo et al. [15, 16]. Responses were divided into three categories based on how they felt about the wild animal: significant problem (negative perception), no problem (positive perception), and no reaction = neutral. The Likert scale [28] states that each attitude statement is expressed as a five-point scale based on how strongly each participant agrees with the statement.

To avoid exaggeration by respondents, field observation of crop loss and livestock depredation was mainly used to confirm the respondent’s response and gather relevant information [36]. According to Yirga and Bauer [51] and Leta et al. [27], the amount of damage caused by wild animals to crops and cattle was calculated based on the tooth marks they left on the damaged plant parts, killed or damaged livestock, and feces. For every type of crop sample, eight two-by-two-meter plots were established on a 1000-square-metre farm. The observation was conducted on representative farmlands in each of the four villages between 2020 and 2021. Every day, observation was done when the seedlings were germinating. Still, the timing of flowering and maturation received more attention. Two observations per week were made in each of these stages. During the visit, each farmland’s complete damaged crop was tallied and recorded on the same day. Damaged plants were added and valued after every developmental stage. Finally, using the work of Yihune et al. [49], the average damage to crops was quantified in kilograms, and the totals were added together and summarized by comparing the current market price and the total yield loss of each village. Similarly, market prices (Ethiopian birr) from the closest town were used to determine each respondent’s financial loss from livestock killed by predators. The results were then converted to US dollars for the various kinds of livestock [20].

### Data analysis

Descriptive and inferential statistics were the main components of data analysis. SPSS version 20 was used for analyzing the data that had been collected. The study used nonparametric χ2 tests to assess the observed prevalence of predation on different livestock kinds across villages, the respondents’ attitudes towards problematic animals, and the seasonality of livestock depredation. To determine the association between agricultural loss, livestock depredation, and village distance from the forest, a correlation was also carried out. The difference between the mean extent of crop raiding and the distance of farmland from the forest edge, the significant variation in the total number of domestic animals killed among the villages, and the significant correlation between the number of domestic animals killed and the seasons were also determined using an ANOVA test. The level of agricultural damage caused by wild animals and the distance of cropland from the forest boundary was tested using Pearson correlation. The profiles of the respondents’ socioeconomic and demographic backgrounds were analyzed using descriptive statistics expressed as frequencies and percentages. Furthermore, descriptive statistics were employed to examine the methods of predation control used by the locals. The statistics were used to determine mean values, ranges, percentages, and frequencies. Every statistical test employed a two-tailed design with a significance threshold of *p* ≤ 0.05. The results were presented using tables and bar graphs.

## Results

### Demographic characteristics of the respondents

**Fig. 2:**
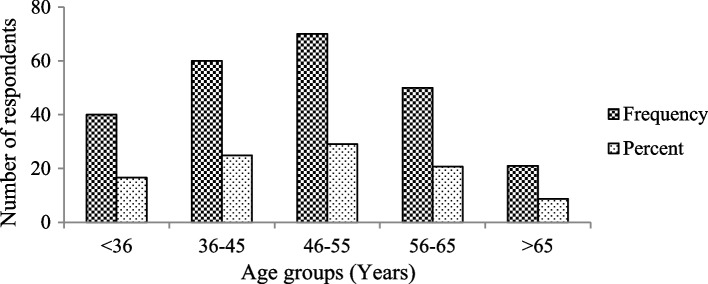
Age group of study participants near the Mankira Forest in the Southwest Region of Ethiopia

Out of the entire sample size, 90.04% were male participants. Female respondents made up 9.96% of the total. There was a significant difference between sex groups (male and female) in the study area (χ^2^ = 64.0, df = 1, *p* < 0.05). The majority of the participants (29.1%) fell within the age range of 46–55 (Fig. 2). Among the respondents, 47.7% were illiterate (cannot read and write), 34.9% had informal education (can read and write), 14.5% attended primary education, and 2.9% attended secondary education.

### Economic activities of the respondents

The primary economic endeavors of the residents in the vicinity of the Mankira forest comprised subsistence farming, encompassing the cultivation of crops, raising livestock, and beekeeping. A total of 212 respondents, accounting for approximately 88%, are involved in mixed agriculture, which includes crop production, livestock rearing, and beekeeping. On the other hand, 15 respondents, equivalent to 6.2%, solely focus on crop production. The remaining 14 respondents, making up 5.8%, rely on crop production along with other labor activities. A statistically significant difference was observed between different economic activities in the study area (χ^2^ = 134.48, df = 2, *p* < 0.05).

**Fig. 3:**
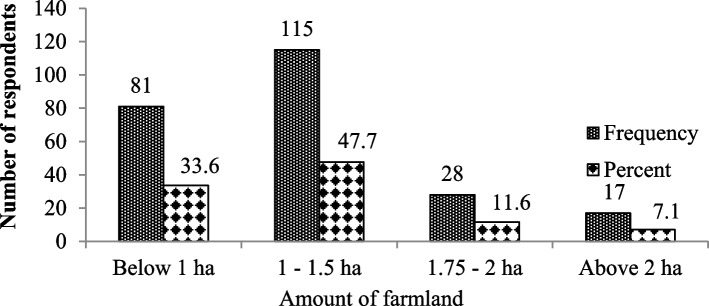
Size of the farmland holdings per household among the respondents near the Mankira Forest in the Southwest Region of Ethiopia

The size of the farmland owned by respondents’ ranged between 0.25 and 3 ha, with a mean of 1.09 ha. Most of the respondents (47.7%) indicated that they have farmland sizes of only 1–1.5 ha. Whereas, 33.6% of the respondents reported that, they have < 1 ha of farm land around the area. Others reported that they have a farmland size of > 2 ha (7.1%) and 1.75–2 ha (11.6%) (Fig. 3).

**Fig. 4:**
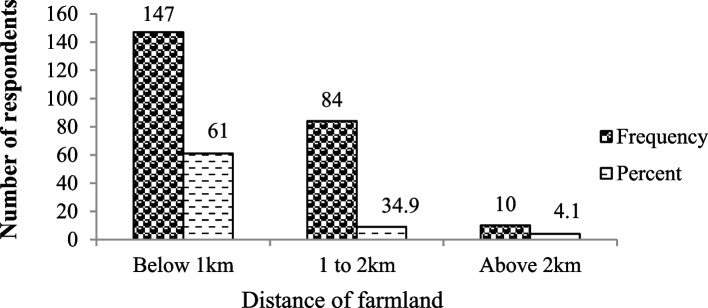
Distance of the respondents’ farmland from the Mankira Forest in Southwest Ethiopia

Most of the respondents (61%) had farmlands within a distance of less than 1 km from the forest edge. Whereas, 34.9% of the respondents had farmland at a distance between 1 and 2 km, and 4.1% of the respondents had farmland at a distance above 2 km from the forest edge (Fig. 4).

The most commonly cultivated crop types around the study area were teff, maize, potato, wheat, and barley. Most of the respondents (49%) cultivate teff and maize. Others cultivated potatoes at 18.2%, wheat at 13.7%, barley at 10.4%, and vegetables and other crops at 8.7% around the forest edge (Table 2). There was a statistically significant difference between different cultivated crops in four villages (χ^2^ = 134.48, df = 2, *p* < 0.05).

**Table 2:** Types of crops mostly cultivated around the Mankira Forest edge by the respondents, Southwest Ethiopia

Types of crops	Villages				Frequency	Percentage
	Gola	Yetiti	Bahita	Becha		
Teff	21	12	20	7	60	24.9
Maize	18	14	17	9	58	24.1
Potato	13	7	10	14	44	18.2
Wheat	3	4	9	17	33	13.7
Barley	2	6	7	10	25	10.4
Vegetables and other crops	12	3	6	0	21	8.7
Total	69	46	69	57	241	100

### The mean number of livestock per household in the villages

**Fig. 5:**
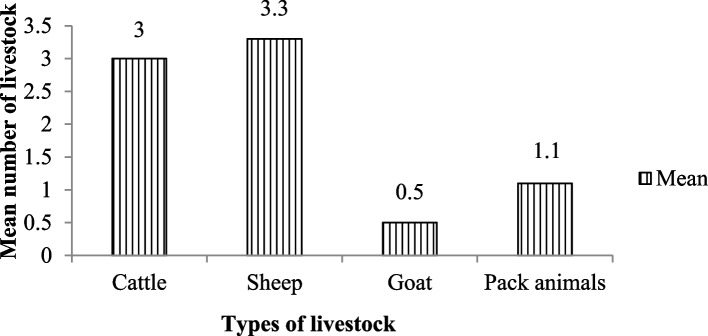
The mean number of different livestock per household in the sampled villages near the Mankira Forest in the Southern Region of Ethiopia

The major livestock kept by the community in the study area were cattle (cow and ox), sheep, goats, and pack animals (donkey and horse). Respondents’ livestock holdings ranged from 2 to 16. There was no significant difference (χ^2^ = 138, df = 9, *p* > 0.05) in the major types of livestock kept in the four villages (Fig. 5). But there was a significant difference (χ^2^ = 8, df = 3, *p* < 0.05) among the total number of livestock owned by each household. In terms of number, the donkey was the least owned per household, while the sheep was the most numerous.

### Crop raiding and the extent of crop raiding caused by the crop raiders

Farmers reported major conflicts with Anubis baboons (39%), Grivet monkeys (24.1%), porcupines (15.3%), rats (13.3%), and birds (8.3%). Respondents place the Anubis baboon as the top crop raider compared to others (Table 3). The amount of crop raiding caused by crop raiders varied statistically significantly throughout the four villages (χ2 = 29.3, df = 4, *p* < 0.05).

**Table 3:** Crop raiding species in the Mankira Forest, Southwest Ethiopia

Species	Scientific name	Frequency	Percentage
Anubis baboon	*Papio anubis*	94	39
Grivet monkey	*Chlorocebus aethiops*	58	24.1
Porcupine	*Hystrix cristata*	37	15.3
Rodents (rats)	Unidentified	32	13.3
Birds	Unidentified	20	8.3

The findings from the participants indicated that crop raiders do not impact all crops in the same way. Among the crops, maize was found to be the most vulnerable to crop raiders, with 115 respondents (47.7%) reporting its susceptibility. Following maize, potatoes (25.3%), wheat (12.5%), and barley (8.3%) were also targeted by crop raiders. However, Teff was identified as the crop least affected by these raiders (6.2%) (Table 4). Crop raiding on different crop varieties was statistically significant in four villages (χ2 = 59.9, df = 4, *p* < 0.05).

**Table 4:** Crop destruction by crop-raiders near the Mankira Forest, Southwest Ethiopia

Crop type	Frequency	Percentage
Maize	115	47.7
Potato	61	25.3
Wheat	30	12.5
Barley	20	8.3
Teff	15	6.2

Maize is the primary crop consumed by the Anubis baboon throughout its various growth stages. Alongside the Anubis baboon, there are other crop raiders such as the grivet monkey, porcupine, rodents, and birds, which cause damage to different crops during different stages of growth. Grivet monkeys damaged maize and barley close to reaching maturity stage during both morning and evening hours. Rodents, particularly porcupines, caused damage to maize and potatoes during the maturation stage, mainly at night. In addition, different species of birds damaged maize, wheat, teff, and barley at the maturation stage of crops during the day (Table 5). Thus, farmers whose farms are situated in close proximity to the forest face a potential threat of losing various crops throughout the entire production season.

**Table 5:** Crop raiders, types, and stages of crops damaged within specific times of the day near the Mankira Forest

Crop raiding animals	Type of crop	Stages of crop raiding	Damage hour of the day
Anubis baboon	Maize	in all stages	evening and morning
	Barley	at fruiting	evening and morning
	Wheat	at fruiting	evening and morning
Grivet monkey	Maize	at seedling	morning and evening
	Barley	at seedling	morning and evening
Porcupine	Potato	at fruiting	at night
	Maize	at fruiting	at night
Rats	Wheat	in all stages	day and night
	Maize	in all stages	day and night
Birds	Wheat	at fruiting	evening and morning
	Maize	at fruiting	evening and morning
	Barley	at fruiting	evening and morning
	Teff	at fruiting	evening and morning

There was a significant difference between the distance of farmland from the forest edge and the mean extent of crop raiding by the crop raiders (*F* = 84.966, df = 2, *p* < 0.05). The Pearson correlation indicated that the distance of the farmland from the forest edge and the mean extent of crop raiding by crop raiders have a slightly negative correlation (*r* = -0.645, *p* < 0.05). The distance of the farmland from the forest edge was an important factor in determining the degree of crop raiding. Based on that, most of the damage was severer around the buffer zone than for farmers whose farmlands are far from the forest edge. Farmers who have farmlands (100%) within 1 km of the forest reported severe crop loss.

### Livestock predation

**Fig. 6:**
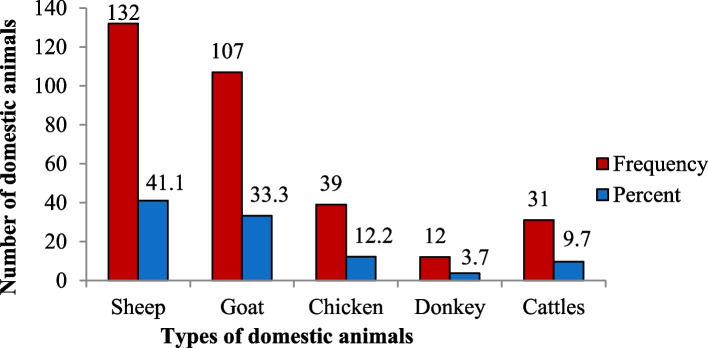
Number of domestic animal losses near the Mankira Forest, Southwestern Ethiopia (between January 2017 and May 2021)

Like the crop loss, domestic animal predation by carnivorous animals was reported from the four study villages. Cattle, sheep, goat, donkey, and chicken losses by the common jackal, spotted hyena, and different kinds of raptor bird species were recorded. According to the feedback provided by participants over the last five years, from January 2017 to May 2021, a sum of 321 predatory assaults was documented. From these, 41.1% were sheep (*Ovis aries)*, 33.3% were goats *(Capra hircus)*, 12.1% were chickens *(Gallus domesticus)*, 3.7% were donkeys *(Equus africanus asinus*), and the remaining 9.7% were cattle (Fig. 6).

There was no significant difference among the villages in the total number of domestic animals killed (*F* = 0.110, df = 3, *p* > 0.05). There was, however, a significant relationship between the seasons and the number of domestic animals killed (*F* = 6.124, df = 1, *p* < 0.05). Livestock predation was higher in the wet season than in the dry season. Of the total of 321 domestic animals killed by predators in the past five years, 67% were killed during the wet season and 33% during the dry season.

The most common predatory wild animal species in the study area was the common jackal, which killed sheep and goats while they were grazing on pasture land during the day. During the late evening, dusk, and night, spotted hyenas have also killed donkeys, sheep, and cattle. During the day, various raptor bird species attack the village chickens (Table 6).

**Table 6:** Types of predator species and the number of domestic animals predated during the last five years around Mankira Forest in Southwest Ethiopia (Archive, Charts of Mankira Keblee, 2021)

Types of predators	Types of livestock lost	Number of livestock killed	Percentage
Common Jackal	Goat, and sheep	187	58.3
Spotted hyena	Cattle, donkeys, and sheep	95	29.6
Raptor bird species	Chicken	39	12.1
Total		321	100

According to the Decha district agricultural office, the amount of domestic animal predation by carnivorous animals increased over time within the Mankira Forest (Table 7).

**Table 7:** Number of domestic animals lost by predators around the Mankira Forest in Southwest Ethiopia (Archive, Charts of Mankira Kebele, 2021)

	Years					Total	Loss in Birr
Livestock type	2017	2018	2019	2020	2021		
Sheep	16	24	26	31	35	132	198,000
Goat	14	20	25	20	28	107	117,700
Chicken	4	5	7	9	14	39	9750
Cattle	3	2	5	8	13	31	248,000
Donkey	1	1	2	3	5	12	72,000
Total	38	52	65	71	95	321	645,450

### Attitude of the respondents towards wildlife conservation

**Fig. 7:**
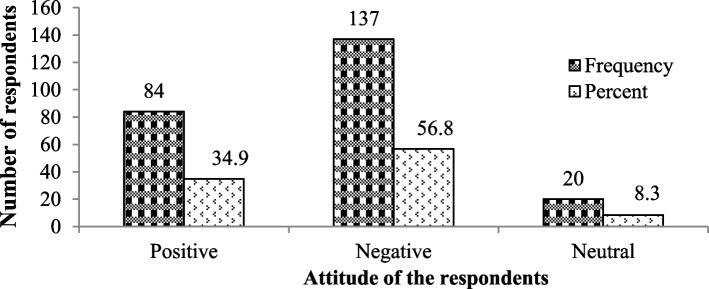
Attitude of the respondents towards wildlife and the forest

Based on the feedback provided by participants, most (56.8%) of the respondents, i.e., 28.5% from Gola, 18.2% from Yetiti, 30.7% from Bahita, and 22.6% from the Becha villages, reported that they had a negative attitude towards wildlife conservation. The main reason given for the latter view was the conflict with wild animals and the resulting economic losses. While 34.9% of the respondents, i.e., 29.8% from Gola, 19% from Yetiti, 29.8% from Bahita, and 21.4% from the Becha villages, reported that they had positive attitudes towards wildlife conservation. Others, including five (25% of respondents) from Gola, five (25% of respondents) from Yetiti, two (10%) from Bahita, and eight (40% of respondents) from Becha villages, reported neutral attitudes towards wildlife conservation (Fig. 7).

The attitude towards wildlife conservation did not vary significantly among respondents with different levels of education (χ2 = 4.257, df = 6, *p* > 0.05). Nonetheless, there was a significant difference in the attitudes of the respondents among the age groups (χ^2^ = 165.019, df = 8, *p* < 0.05). Most of the respondents, 84.5% with an age group above 56 years old, have a positive attitude towards wildlife and forest habitat (Table 8). The Spearman correlation coefficient (*r* = -0.427, *p* = 0.000) also showed that there was a significant negative correlation between the age group of the respondents and their conservation attitude towards the wildlife around the Mankira forest.

**Table 8:** Attitudes of the respondent s’ among different age groups towards wildlife conservation

Attitude	Age group					Frequency	Percent (%)
	< 36 years	36–45 yrs	46–55 yrs.	56–65 yrs	> 65 yrs.		
Positive	6	11	7	45	15	84	34.9
Negative	25	49	63	0	0	137	56.8
Neutral	9	0	0	5	6	20	8.3
Total	40	60	70	50	21	241	100

## Discussion

The study’s findings indicate that the farmers who live close to the Mankira forest suffered harm or were impacted by wild animals. Given that the majority of the population makes their living from agriculture, the issue is more serious. Seven of the most significant wild creatures that impact the Mankira forest’s farmers’ livelihoods through crop raiding and livestock predation have been identified. These included the spotted hyena, common jackal, porcupine, anubis baboon, grivet monkey, and raptors. Several scholars identified those animals as significant agricultural pests in several regions of Ethiopia, citing them as important livestock predators and crop raiders [17, 35, 44, 45, 47]. Crop raiding becomes the primary issue influencing locals’ livelihoods in rural areas where agriculture is essential to their survival [19, 26, 25, 46]. According to Gobosh et al. [18], the southwest Ethiopian Gera District was experiencing issues with livestock predation as well as crop destruction.

The interviewees provided a range of reasons for the diverse impacts of wildlife in the Mankira forest. The majority of respondents concurred that the main factors contributing to the impact of wildlife on the local population were agricultural expansion and deforestation for the purposes of gathering firewood and expanding farmlands. This discovery aligns with the research conducted by Derebe et al. [10] and Bashyal et al. [4], indicating that conflicts emerge in forested areas between humans and wildlife species as a result of resource competition. Likewise, an investigation carried out in the Gera district of southwest Ethiopia revealed that the primary reason for the effects of wildlife on the populace was the growth of agriculture near the forest boundary [18].

Depending on the kind of crop planted and the kind of wild animal engaged in crop raiding, different crops were more or less vulnerable to damage caused by wild animal pests[18, 35]. Recent research revealed that crop raiding was worse in villages close to the forest, such as the Bahita and Gola villages, than it was in areas farther away from the forest’s edge. It is well known that crop raiders typically target farms near wild animal habitats, making them more susceptible to harm [6, 30]. Bezihalem et al. [6] found that the proximity of cropland to the forest boundary is directly correlated with the extent of damage caused by crop raiders. Costs associated with farming close to forests include crop loss, livestock predation, and time and money lost trying to protect the crops and livestock [6, 7, 29, 45].

Crop raiders did not harm all crops in the study area equally. The crop type that was most susceptible to crop raiders in the current investigation was maize. Its high farm sizes relative to other crops in the area and nutritional values [41] could be the causes. In contrast, the majority of respondents in research carried out in Choke Mountain, Ethiopia, stated that potatoes were the crop in the region that was most susceptible to crop raiders [6]. Wheat and barley were the most susceptible crops to invaders, according to Mekonen [35].

When it comes to the main predators that cause problems in the study villages, the majority of respondents stated that various predators, such as spotted hyenas, common jackals, and several raptor bird species, had taken goats, sheep, chickens, donkeys, and cattle within the previous five years. This result is consistent with the findings of Derebe et al. [10], Dar et al. [9], and Biset et al. [7], which found that predators such as common jackals and leopards, black bears, red fox Asiatic, and wolves may pose a threat to local residents in the areas surrounding Dachigam National Park in Kashmir, India; Banja Woreda, Awi Zone, Ethiopia; and Borena Sayint National Park in northern Ethiopia. This result is consistent with earlier research conducted in several regions of Ethiopia [15, 16, 35]. The amount of damage caused by wildlife and the village’s proximity to the forest were significant determinants of the livestock losses brought on by the predators. Across the nation, a study of a comparable nature has also been published Biset et al. [7]; Derebe et al. [10].

Though there are rare exceptions, cattle predation generally follows seasonal patterns, according to Holmern et al. [21] and Dar et al. [9]. Livestock predation was highest during the wet season and lowest during the dry season in the current study. This finding is consistent with research by Megaze et al. [34] in Ethiopia’s Chebera Churchura National Park, which found that almost 56% of all predation incidents occurred during the wet season. This was comparable to observations made in Kenya’s Tsavo National Park [40]. In Cameron’s Waza National Park, a similar discovery was made (Bauer [5]). This may be connected to both adequate habitat cover and seasonal variations in the wild prey’s distribution. However, research done in Ethiopia’s Guassa Mountain revealed that most predation incidents happened during the dry season. The low natural abundance of rodents at this time may be the cause of the higher intensity [3]. According to the current study, there may be a higher risk of livestock predation if goats and sheep in particular are left unattended throughout the day.

According to the majority of respondents, there has been a periodic increase in trends in wildlife impacts, such as damage to crops and livestock predation. The findings were in line with research carried out in the Gera district of Ethiopia by Gobosh et al. [18], in the Choke Mountains by Bezihalem et al. [6], and in the Dachigam National Park area of Kashmir, India, by Dar et al. [9]. Additionally, studies carried out in Wolaita Sodo Zuria, Ethiopia, by Kebede et al. [23] showed that during the previous five years, crop raiders’ damage to crops increased.

The results indicate that respondents’ opinions about wildlife conservation varied significantly depending on the kind of animals. The vast majority of respondents had negative attitude about protecting wildlife. This result is consistent with the majority of research conducted in developing nations, where the local community, as reported by Mkonyi et al. [37]; Marneweck et al. [31]; Merkebu and Yazezew [36]; Gebo et al. [15, 16], wants the carnivore population to decline since it is causing damage to their livestock.

Gebo et al. [15, 16] stated that two-thirds of respondents opposed the conservation of carnivores in the human-dominated area of southern Rift Valley, particularly spotted hyenas, black-backed jackals, common genets, and mongoose species, because they are the primary cause of livestock depredation, while supporting the conservation of lions, caracals, African civets, and leopards. In a comparable manner, 59.71% of respondents to research by Kebede et al. [23] in the Wolaita Sodo Community Forest, Ethiopia, expressed negative attitudes about the animals that were causing problems. Comparably, a survey carried out in Ethiopia’s Borena Sayint National Park revealed that the majority of households had negative attitudes towards the conservation of wildlife [7]. However, the majority of the population (74.8%) that resides in the vicinity of Semien Mountain National Park had positive attitudes towards wildlife and conservation [49].

According to Eshete et al. [13], even slight amounts of livestock predation may make people feel negatively about wildlife, which could have a big impact on the conservation of wildlife in that area. Respondents who were in favour of wildlife cited several reasons, including the fact that it preserves the environment, draws tourists, offers sustenance during times of extreme food scarcity, is visually beautiful, and will be vital for future generations. According to Matusal et al. [33], respondents who had negative attitudes towards wildlife species observed wild animals as possible crop raiders, livestock depredators, disease carriers, and threats to humans. Conversely, respondents who had neutral attitudes did not offer any explanation for their lack of concern for wildlife. According to Araya et al. [2], the absence of community participation in benefit sharing and decision-making has resulted in indifference and an attraction for forced access to resources inside enclosures, which frequently leads to conflict. The respondents’ attitudes towards the conservation of wildlife were influenced by both their age and the severity of the damage that wild animals caused. For these reasons, the majority of respondents (80.6%) stated that most of the individuals who were found close to the forest had a negative attitude towards the conservation of wildlife. In the research area, there was a growing tendency towards livestock depredation. The amount of livestock that has been predated is predicted to rise over the next ten years, as evidenced by the increasing rate of predation trend estimation across communities. Over 50% of the participants emphasized the growing patterns of livestock predation. Above half of the respondents (63.5%) stressed the increasing trends of livestock depredation. The study indicated that those who lived furthest away from the forest felt relatively better about animals than those who lived closer to it. This proved that a community’s attitude towards animals was impacted by how far away the animals were from their natural habitat. This could result in fewer people living beside wild animals in the study area and the emergence of negative perceptions about them.

Given that community support and participation are essential to the success of conservation activities, views about wildlife conservation in Ethiopia have a big impact. The success or failure of Ethiopia’s efforts to conserve wildlife is greatly influenced by the attitudes of the community. Strategies for conservation are more effective when there is a positive attitude, active participation, and community empowerment. Thus, in order to accomplish long-term conservation goals, it is imperative that conservation practitioners and policymakers give community engagement top priority and foster strong relationships with local residents.

## Conclusion and recommendations

Farmers in the study area reported that livestock theft and crop raiding by wild animals significantly hindered their livelihoods, with incidents being more frequent near forest edges. Key culprits included raptors, jackals, hyenas, porcupines, baboons, and monkeys, with jackals primarily responsible for livestock predation and baboons for crop damage. Consequently, most farmers held negative attitudes towards wildlife conservation due to the damage caused. However, some acknowledged the forest’s value despite these challenges. The study suggests that the collected data on crop raiding and livestock predation can serve as a baseline for developing wildlife management plans aimed at fostering sustainable coexistence between local populations and wildlife. It recommends creating a conservation strategy that benefits both farmers and wildlife and forming the basis for further research on wildlife ecology, conservation, and community-based practices. To achieve sustainable coexistence, enhancing community benefits from the forest is crucial. Practical measures, such as collective guarding efforts, are recommended to protect crops and livestock. The study highlights the need to address several gaps in understanding and managing human-wildlife conflict through research on predation, raiding, and community attitudes. These efforts are essential for informing effective policy and management strategies. Overall, this study bridges the gap between conservation theory and practice, promoting shared ecosystem management that benefits both people and wildlife.

## Data Availability

“All data generated or analysed during this study are included in this published article.”
